# A Design for Additive Manufacturing Strategy for Dimensional and Geometrical Quality Improvement of PolyJet-Manufactured Glossy Cylindrical Features

**DOI:** 10.3390/polym13071132

**Published:** 2021-04-02

**Authors:** Natalia Beltrán, Braulio J. Álvarez, David Blanco, Fernando Peña, Pedro Fernández

**Affiliations:** IPF Research/ARAMO Group, University of Oviedo, 33203 Gijón, Spain; nataliabeltran@uniovi.es (N.B.); braulio@uniovi.es (B.J.Á.); dbf@uniovi.es (D.B.); penafernando@uniovi.es (F.P.)

**Keywords:** design for additive manufacturing, quality enhancement, material jetting, features of linear size

## Abstract

The dimensional and geometrical quality of additively manufactured parts must be increased to match industrial requirements before they can be incorporated to mass production. Such an objective has a great relevance in the case of features of linear size that are affected by dimensional or geometrical tolerances. This work proposes a design for additive manufacturing strategy that uses the re-parameterization of part design to minimize shape deviations from cylindrical geometries. An analysis of shape deviations in the frequency domain is used to define a re-parameterization strategy, imposing a bi-univocal correspondence between verification parameters and design parameters. Then, the significance of variations in the process and design factors upon part quality is analyzed using design of experiments to determine the appropriate extension for modelling form deviation. Finally, local deviations are mapped for design parameters, and a new part design including local compensations is obtained. This strategy has been evaluated upon glossy surfaces manufactured in a Vero™ material by polymer jetting. The results of the proposed example showed a relevant improvement in dimensional quality, as well as a reduction of geometrical deviations, outperforming the results obtained with a conventional scaling compensation.

## 1. Introduction

Additive manufacturing (AM) processes make parts from 3D model data, usually layer upon layer [1]. The theoretical geometry of a three-dimensional object is sliced into bi-dimensional shapes that are later manufactured and stacked vertically. Industrial adoption of AM is still hampered by an insufficient mass production capacity, a limited range of processable materials, and a lower manufacturing quality than achievable through alternative conventional processes [2]. There is a gap between AM specification standards and industrial requirements [3] that demands greater efforts in the fields of standardization, offline verification, on-machine measurement, and process control. In this sense, the evaluation of dimensional and geometrical quality in additive manufacturing of polymeric parts has been the subject of extensive research.

Some research studies [4,5,6] were focused on characterizing the process capability from a metrological point of view. Different approaches range from using a gage repeatability and reproducibility (G&R) methodology for the capacity evaluation of a polymer jetting system [5], to benchmarking comparisons of dimensional and geometrical accuracy between different polymer AM processes [6]. Other studies [7,8,9,10,11,12,13,14,15] analyzed the influence of design, process, and production factors on part quality. Part size, location, and orientation are among the most frequent factors [8,9,12,13], whereas inner design factors and process factors also have an influence upon quality results [10,14,15]. These works described part quality in metrological terms by means of the differences between nominal and actual sizes, the compliance of geometrical tolerances, or alternative quality indicators like the volumetric error.

Nevertheless, the deviation of a manufactured part from its theoretical shape cannot be fully described by means of a single parameter, like size error, flatness, or cylindricity, but demands an effort to model geometrical deviations. This necessity has been addressed under different points of view, and modelling of geometrical distortion has been frequently used in simulation of part quality assessment, simulation of assembly feasibility, or form errors compensation. Henke et al. [16] modelled the form error of cylindrical features with different methods like Chebyshev polynomials, Fourier series, and Eigen shapes. They pointed out the relevance of selecting an optimal measurement strategy to characterize the form errors with the minimum number of points. Other researchers [17,18] used the discrete cosine transformation to model geometric errors allowing for the identification of different error pattern sources, including part position and orientation. The “skin model” concept [19] was adapted by Schleich et al. [20] to develop skin model shapes as finite representations of skin model. Skin model shapes are more suitable for computing and simulation, and they have been extensively used for tolerance analysis [21], assembly simulation [22,23], or contact modelling [24]. Different sources of form deviation could be modelled by skin model shapes, ranging from machine-related modes [25], design files format and material shrinkage [26], or position and orientation [24], which makes them suitable for assist design for additive manufacturing (DfAM approaches) [27]. 

Geometrical deviation modelling has therefore been used to increase the comprehension of the relationship between process parameters and manufacturing errors. It has been a key tool for assembly simulation and tolerance analysis and fundamental to a series of works aimed at the improvement of dimensional and geometrical quality through the modification of the input geometry. Tong et al. [28] described form errors in material extrusion (MEX) processes as a function of 18 parametric errors and elaborate compensation models for the STL file, and for individual slices, achieving clear improvements on part geometry. The group of Professor Huang has done extensive research in shape deviation modelling and compensation [29,30,31,32,33,34,35,36]. They proposed a polar deviation model, especially suited for cylindrical surfaces, where the deviations between the actual surface and the nominal one were evaluated in the normal direction to each surface point [29]. The proposed methodology was used for shrinkage compensation in a stereolithography process. They pointed out the inconveniences of discrete approaches with many parameters (STL or finite element analysis compensations) related to the balance between deviation acquisition accuracy and computational complexity. The model was extended to a system-level approach to predict and compensate generic shapes [30] and used to evaluate the effect of a discrete compensation using a finite number of levels [31]. Their model has been also extended to generalized shapes [32] and previously untried shapes [33] to create a global deviation modelling strategy. Additionally, they have made attempts to extend 2D compensation to 3D compensation by dividing the problem into in-plane deformation (concerning the cross-section) and out-of-plane deformation [35,36]. Deviation models have sometimes been obtained from large amounts of data [37,38,39], especially when 3D optical scanning instruments were used for geometrical characterization of parts, but also when models obtained through finite elements analysis [38,40] were used. Some of these works proposed a pre-distortion of the input files to compensate for form errors, which has been highlighted as a relevant strategy for quality control in AM [41]. 

According to the current state of art, geometrical deviations are not usually considered at the design step. Similarly, it is not frequent to impose a direct correspondence between design parameters and verification parameters. In those applications that used an STL file as a reference, an interpolation process was applied to determine the position of each STL vertex from the digitized point-cloud [37], and this is a consequence of the lack of a direct correspondence between both design and verification sets of points. In some works [42] the digitized point cloud needs to be rasterized first and transformed into a regular grid to improve the comparison with the nominal geometry. Even in those cases where the reference geometry has been already parameterized to model geometrical distortion [40], a fitting procedure between the information from distorted geometry and the original geometry was carried out. Moreover, some works [12,39] showed that parameters related to production planning, like the position or orientation of a given part within the manufacturing space, could have an influence upon the modelling of geometrical deviations. Nevertheless, most compensation models did not usually take this fact into account. Finally, despite the huge effort that has been conducted to improve manufacturing quality in AM, it is surprising that many of these works share a vision of geometrical quality as a stand-alone goal, without considering the actual relevance of the objective from a functional point of view. Although the overall objective of improving the similarity between the theoretical design and the manufactured parts is relevant, there are many situations where, despite the deviations caused by the manufacturing process, the manufactured geometry is perfectly suitable for the design purpose and does not need to be improved. In fact, most commercial systems are perfectly capable of achieving quality levels that fit in the range of general tolerances [43], even in the cases of low-cost MEX printers [44]. Accordingly, special attention must be paid to those features that could be subjected to highly restrictive dimensional tolerances as well as to geometrical tolerance requirements, which are mainly those involved in fittings: Cylinders or pairs of parallel opposite surfaces [45]. These features of linear size (FoLS) [46] are characterized because they contain opposed points, they have a reproducible derived median point, axis, or center plane, and they have a limited extension.

In a previous work, Beltrán [47] proposed a systematic framework for fulfilling dimensional and geometric tolerances of FoLS in AM processes. The proposed methodology was divided in several stages: The characterization of achievable quality, the optimization of process parameters, and the optimization of part design. Process optimization is used to find an optimal combination of process parameters in those cases where this optimal configuration has not been previously determined by machine or material specifications. If quality requirements were not fulfilled after the second stage, a Computer Aided Design (CAD) optimization procedure will be carried out through a re-parameterization of the original design to compensate the geometric distortions. This procedure would be performed using the optimal configuration of process parameters previously determined. 

**Figure 1:**
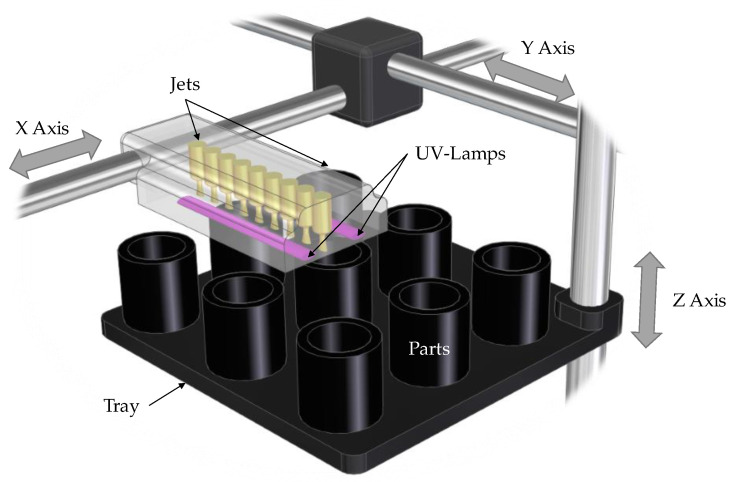
Basic scheme of a PolyJet jetting operation.

The present work focuses on the application of a DfAM approach to optimize “glossy” cylinders manufactured in a Vero^TM^ material with the PolyJet material jetting (MJT) technology (Figure 1). 

In MJT processes, “droplets of feedstock material are selectively deposited”, according to ISO/ASTM 52900 [1]. The PolyJet technology is based on jetting an acrylic photopolymer over flat surfaces to form layers that are then cured with ultraviolet radiation (Figure 1). 

An injection block (or printing head) consists of an array of injection orifices that are capable of independently dispensing certain amounts of material. The injection block moves back-and-forth along the *X* axis whereas a reposition movement along the *Y* axis takes place between consecutive material deposition movements. Droplets are projected onto the workspace to precisely define regions of the model and/or support material within each layer. MJT processes are more accurate than MEX processes. A comparative analysis [48] indicated that, for a set of circular dimensions, an average 0.74% relative error was found when manufacturing in an Objet 30, whereas this percentage increases to a 2.34% in the case of an Ultimaker MEX machine. Variability of measured sizes is also low in Polyjet; e.g., a long-term Process Capability analysis [5] found that the standard deviation value for measured sizes of a nominal 14.5 mm cylindrical glossy surfaces (n = 150) was lower than 0.010 mm. Nevertheless, quality issues are still a matter of concern up to now [5,49,50]. 

Some authors discussed the influence of process parameters upon part quality, so that an optimization could be achieved by selecting the proper configuration [12,51]. Nevertheless, Brajlih [52] pointed out that the “main accuracy problem of the PolyJet technology is shrinking of the building material during the phase of polymerisation”. In fact, the manufacturing software used in MJT machines incorporates independent scale factors in each axis to allow direct application of compensation factors. Brajlih used genetic programming to relate mathematically nominal sizes and manufactured sizes to model scale factors as size-dependent parameters. This scaling strategy was later used to manufacture optimized parts. Accordingly, a shrinkage compensation strategy seems to be the best option for accuracy improvement of FoLS in a PolyJet MJT technology.

Notwithstanding the current state of art, the present work proposes an alternative geometric deviation modelling and compensation strategy tailored to improve dimensional and geometrical accuracy of parts manufactured with the PolyJet technology. The DfAM strategy presented here is based on four premises:

- Geometrical re-parameterization shall be carried out upon the design file, so that it could be implemented using a conventional 3D design software package.
- A suitable verification strategy shall be determined to obtain a bi-univocal correspondence between design parameters and verification parameters.
- Compensation models shall be defined after checking how dimensional and geometrical quality is influenced by design, process, or production parameters that were not included in the recommended process configuration.
- The proposed strategy shall be oriented to medium-to-large production batches, and experimental effort shall be kept to a minimum.

The paper is organized as follows: Section 2 describes the DfAM approach. An application example is provided in Section 3, where the obtained results are also presented and discussed. Relevant conclusions are finally summarized in Section 4.

## 2. Description of the Proposed DfAM Strategy

**Figure 2:**
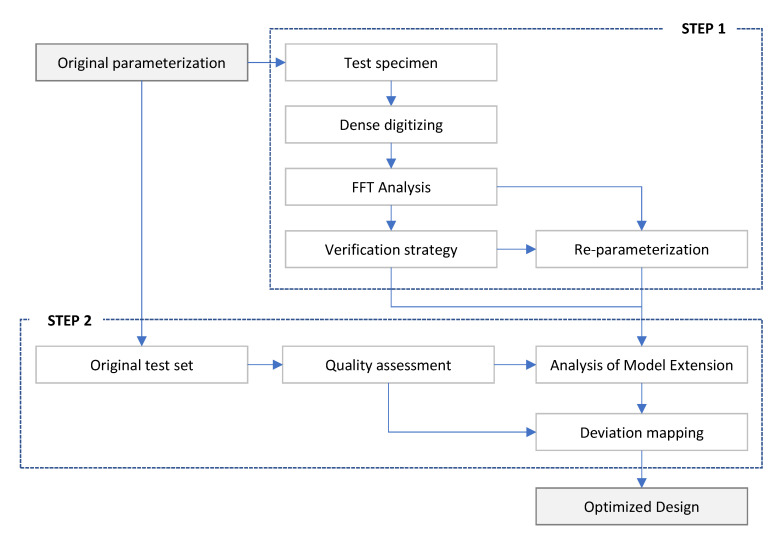
Flowchart of the proposed design for additive manufacturing (DfAM) optimization strategy.

The proposed strategy (Figure 2) has been structured considering two consecutive steps.

- Selection of a proper verification and re-parameterization strategy.
- Deviation modelling and design optimization.

A detailed description of the different tasks involved in each step is provided bellow.

### 2.1. Step 1: Selection of a Proper Verification and Re-Parameterization Strategy

**Figure 3:**
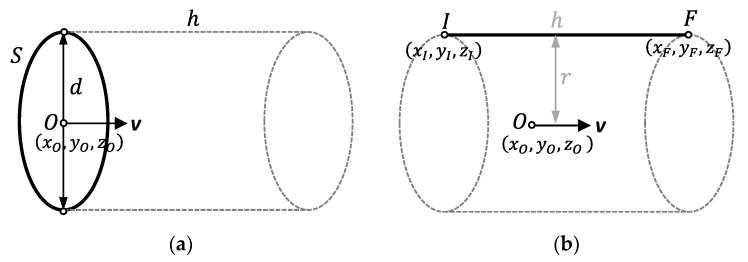
Usual cylinder parameterizations in CAD systems: (**a**) Extrusion of a circumference; (**b**) revolution of a generatrix.

Following Henke et al. [16], this first step defines an optimal verification strategy for characterizing form errors with the minimum number of points. The proposed DfAM approach establishes a bi-univocal relationship between verification points and design parameters. An evaluation test specimen shall be manufactured and digitized using the original parameterization, to perform an initial analysis of form deviations. In the case of right circular cylinders, CAD designers usually employ two alternative parameterizations to define the boundaries that enclose the solid: extrusion of a circumference or revolution of a generatrix (Figure 3). In an extrusion operation, a planar cross-section is extruded a distance (*h*) along a straight direction (***v***). Most CAD systems have a specific command to generate orthogonal extrusions, so that the normal to the plane that contains the cross-section defines the extrusion direction. In the case of right circular cylinders, the cross-section is a circumference defined by the location of its center (*O*) and its radius (*r*) or diameter (*d*). 

In a revolution operation, the boundary that encloses the solid is generated by the revolution of a line around an axis or spine. Right circular cylinders can be therefore generated by revolting a straight line around an axis, being both parallel. The parameterization is given by the direction (*v*) and the location (*O*) of the axis and the coordinates of the initial (*I*) and final (*F*) points of the straight line, given the radius (*r*) as the distance between the line and the axis of revolution. Both parameterization approaches can be used to generate surfaces or solids, depending on the design mode. They control the size of the cylindrical feature and its location and orientation with respect to a coordinate system, whereas the shape of the feature is a mathematically exact cylindrical form. 

Once the test specimen has been manufactured, its shape shall be digitized using a dense sampling strategy [16], which would allow for an adequate analysis of shape deviations. In the case of a right circular cylinder, this could be achieved using a bird-cage extraction strategy [53]. Consequently, discrete surface points shall be digitized along generatrixes and cross-sections. Axial section planes will be used to define generatrixes in different orientations, while cross-sections will be contained in planes perpendicular to the cylinder axis. This digitizing strategy allows for independent descriptions of several generatrixes and cross-sections along the surface of the cylinder. 

The proposed strategy adapts the recommendations contained in ISO 12180-2:2011 [53], regarding the relationship between surface decomposition into several sinusoidal components and the appropriate sampling strategy for quality assessment. Accordingly, each individual set of data shall be treated as a discrete signal and decomposed by means of the fast Fourier transform (FFT). In the case of a cross-section extracted from a cylinder and resembling a circumference, the fundamental wavelength of the Fourier series would be the length of that circumference: One undulation per revolution (UPR). On the other hand, the objective of the analysis here is to highlight relevant components that significantly contribute to shape deviation. Accordingly, high frequency components will presumably have a reduced interest. In fact, ISO 12181-2:2011 [54] recommends the use of longwave pass filters, although they have some drawbacks, like distorting waviness content or not-fully suppressing roughness content. 

We propose that an analysis of significance of each Fourier component contribution to total distortion shall be driven in a first stage. The analytical sequence firstly determines a reduced set of frequencies that show a significant amplitude in all samples of the same type (cross-sections/generatrixes). Then, the highest frequency corresponding to a relevant signal component (*f_c_*) shall be independently determined for cross-sections and generatrixes. Later, the Nyquist criterion shall be applied to calculate a minimum sampling frequency (*f_s_*) that should be higher than twice *f_c_*. Consequently, the period between sampling points in a given profile should fulfil in Equation (1).

(1) $$T_{s}<\frac{1}{2\cdot \;f_{c}}$$

Two independent sampling periods shall be calculated for cross-sections (*T_c-s_*) and generatrixes (*T_g_*). Accordingly, the minimum number of verification points to be digitized in each cross-section (*n_c-s_*) and generatrix (*n_g_*) shall be determined. 

**Figure 4:**
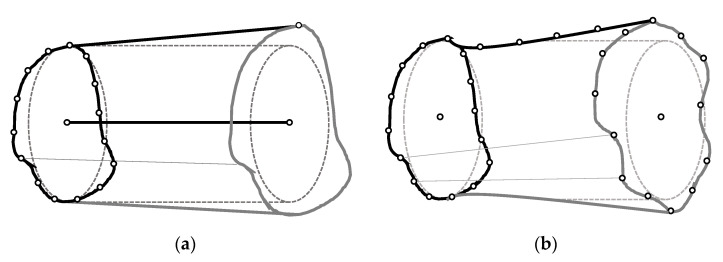
Examples of alternative parameterizations for a deformed cylindrical surface: (**a**) Sweep of a single cross-section with a straight peripheral guide; (**b**) loft of two cross-sections with a curved peripheral guide.

The optimal verification strategy and the re-parameterization of the cylinders are related by means of a bi-univocal correspondence between verification points and design parameters. Since form deviations could not be modelled through conventional parameterization, two main alternative options can be found in most CAD software packages: Sweep operations and loft operations. In sweep operations, a surface is generated by sweeping a cross-section along a spine (Figure 4a). In loft operations, a surface is created in the space between at least two cross-sections with or without a spine (Figure 4b). Both sweep and loft operations could also use peripheral guidelines that influence the shape of the surface in the space between cross-sections. 

In the case of a deformed right cylinder, and depending on the results of the Fourier analysis, the designer could select the most suitable alternative parameterizations, considering the specific CAD system. If *n_c_*_-*s*_ *≫ n_g_*, re-parameterization can be structured as *n_g_* cross-sections with *n_c-s_* verification points. On the other hand, if *n_c_*_-*s*_ *≪ n_g_*, re-parameterization can use *n_c-s_* generatrixes with *n_g_* verification points. Finally, if *n_c_*_-*s*_ *≅ n_g_*, both cross-sections and generatrixes would be simultaneously used in a bird-cage design. In any case, this strategy proposes a spline-based re-parameterization, imposing a univocal correspondence between the control nodes of each spline and the previously defined verification points.

### 2.2. Step 2: Deviation Modelling and Design Optimization

Deviation modelling comprises two concurrent tasks: Quality assessment and analysis of model extension. Quality assessment evaluates the dimensional and geometrical accuracy achievable through a conventional parameterization, whereas analysis of model extension defines how an effective deviation modelling should be implemented, according to different sources of variability. The requisite of minimizing experimental effort led to consider the same data for both tasks.

Quality assessment starts with the selection of quality indicators (QI). In the case of standing-alone cylindrical surfaces, two QI are commonly used: Size and cylindricity. According to ISO 286-1:2010 [45], the size of a cylinder of perfect form (nominal cylinder), as defined by the drawing specification, is denominated “nominal size” (*S_N_*). Moreover, the size of the associated integral cylinder, that is established from the extracted integral cylinder, is denominated “actual size”. The proposed optimization strategy selects the least squares reference cylinder (LSRC) [53] as the associated integral cylinder, in accordance with ISO 14405-1 [46]. Thus, the term “actual size” (*S_A_*) will be used to design the LSRC diameter. Accordingly, size deviation (*ΔS*) could be defined as the difference between *S_N_* and *S_A_* (Equation (2)). The lower *ΔS*, the closer the actual size to the nominal size and the better the achieved dimensional quality.

(2) $$\Delta S=S_{A}-S_{N}$$

Cylindricity, on the other hand, may be characterized by several alternative parameters, like the root-mean square cylindricity deviation or the peak-to-reference cylindricity deviation. The present strategy calculates cylindricity according to the minimum zone reference cylinders as the radial difference between two coaxial cylinders enclosing the actual cylindrical surface and having the least radial separation [53].

For parts containing more than one cylinder, additional QI could be included in both the quality assessment and the analysis of model extension; coaxiality, parallelism between axes, or perpendicularity of the axis with respect to the planar base could be among them. In any case, those QI that were considered relevant for a given part shall be measured according to the defined verification strategy.

Analysis of model extension requires the definition of a shortlist of factors that could have an influence upon the variability of the proposed QI. Manufacturing is frequently conducted under a given configuration of process parameters related to best-practices knowledge. Depending on the process and the specific characteristics of the part that is subjected to the optimization process, possible factors could include design factors (size, relative location of features, or type of feature), process configuration factors (temperature, energy, or speed), or production factors (location or orientation of parts within a manufacturing tray). Design factors are connatural to the part while production related factors are subjected to modifications depending on batch size and machine availability. Process configuration, on the other hand, is usually determined by the recommendations of the manufacturer or the supplier. When using proprietary materials, like is the case of the PolyJet technology, the manufacturer provides a recommended configuration of process parameters. This recommended configuration includes factors like layer height, injector velocity/acceleration or UV lamp intensity. Otherwise, if the systematic framework described in [47] were applied, process configuration should have been optimized before the CAD optimization stage.

The proposed DfAM strategy uses design of experiments (DOE) and the analysis of variance (ANOVA) to evaluate the significance of factors variability upon the variability of QI. Within the scope of the proposed strategy, the objective of DOE is to identify which factors have a relevant influence upon part quality, leading to a particularization of the deviation modelling. In the case that none of the evaluated factors showed relevance, a single deviation model could be used. On the other hand, if there were several significant factors, individual deviation models should be elaborated for each combination of factor levels. The output of this step will be a complete definition of the characteristics of deviation models, regarding individual cylinder parameterization and deviation model extension.

**Figure 5:**
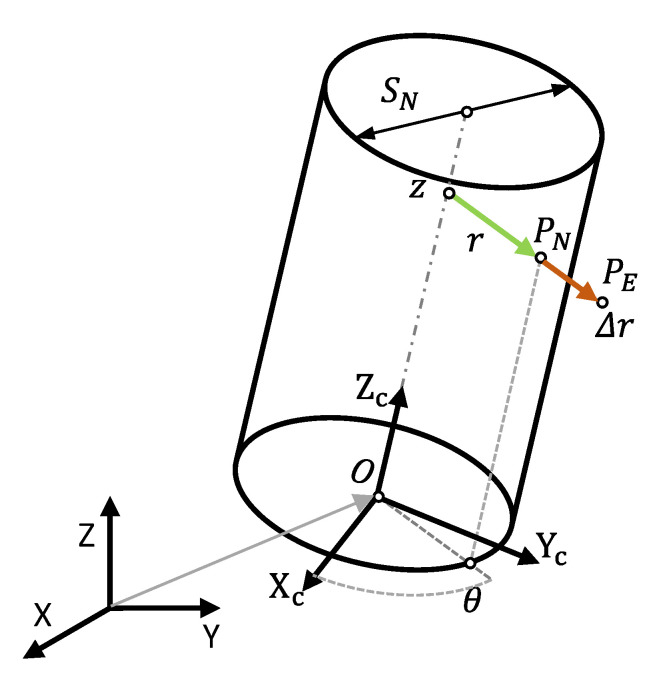
Schematic representation of the radial deviation (*Δr*) between the nominal position of point P (*P_N_*) and its correspondent extracted position (*P_E_*), considering local cylindrical coordinates.

Once model extension has been defined, the local deviation values for all the control nodes in the re-parameterized design will be calculated in a deviation mapping procedure. Deviation mapping could be exclusively based on the results obtained during previous steps, which could contribute to the objective of using the minimum experimental effort or, on the contrary, demand manufacturing additional trays. In the case of cylindrical features, using a polar coordinate system is the best option [35]. Considering the spline-based re-parameterization, the relative position of each node (*P*) could be parameterized by its radial distance to the axis of the reference cylinder (*r*) for a given combination of height (*z*) and azimuth (*θ*) (Figure 5). It must be noted that, in the case of parts that include a coaxiality or parallelism condition, re-parameterization should relate some features to a reference one (datum). Consequently, deviation mapping should consider the relationship between features, so that deviation values can be calculated with respect to the proper reference. Accordingly, deviation mapping uses a reference system that is linked to each feature or to a reference datum (Figure 5).

According to the proposed DfAM strategy, the objective of an ideally exact manufacturing requires the radial distance of each node in the nominal cylinder with respect to the nominal axis to be equal to the radial distance of the corresponding point in the actual (manufactured) cylinder with respect to the LSRC axis. This condition would implicitly assume that the nominal, actual, and LSRC cylinders are identical. Nevertheless, inaccuracies of manufacturing systems will cause each extracted integral cylinder and its associated integral cylinder to deviate from the nominal cylinder. Accordingly (Figure 5), each extracted point (*P_E_*) digitized on the surface of the manufactured cylinder will be displaced a distance (*Δr*) in the normal direction to the nominal surface with respect to the corresponding nominal point (*P_N_*).

**Figure 6:**
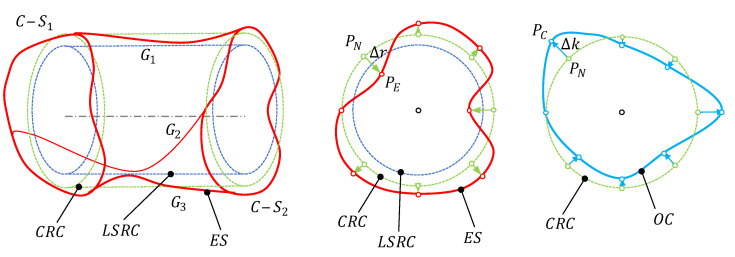
Schematic representation of the deviation mapping and design compensation procedure.

This strategy matches up the axis of the nominal cylinder and the axis of the LSRC cylinder, so that a common framework allows for the characterization of local deviation *Δr*. This calculation shall be performed for all the points included in the verification procedure. Accordingly, the reference for *Δr* calculation shall be a compensation reference cylinder CRC (Figure 6), that is defined by the axis of the LSRC and the nominal size. The re-parameterized design could be improved by applying local compensation coefficients (*ΔK*), whose values shall be calculated from *Δr* measured values. Thus, if the model is constructed from data of several supposedly identical parts, *ΔK* would be calculated as the negative average of individual *Δr* values (Equation (3)).

(3) $$\Delta K=-\frac{\sum_{i=1}^{n}\Delta r_{i}}{n}$$

Figure 6 provides an example of deviation mapping and design compensation. From the data of the extracted surface (ES) obtained during the deviation modelling step, a LSRC is defined. Next, a CRC is built to calculate *Δr* values for each verification point. Each *ΔK* value for a particular point is then calculated from its correspondent *Δr* values (Equation (3)), and the position of a compensated point (*P_C_*) is finally obtained. Repeating this procedure for all nodes in the re-parameterized design, an optimized cylinder (OC) is defined for each combination of relevant factors determined from the ANOVA. 

**Figure 7:**
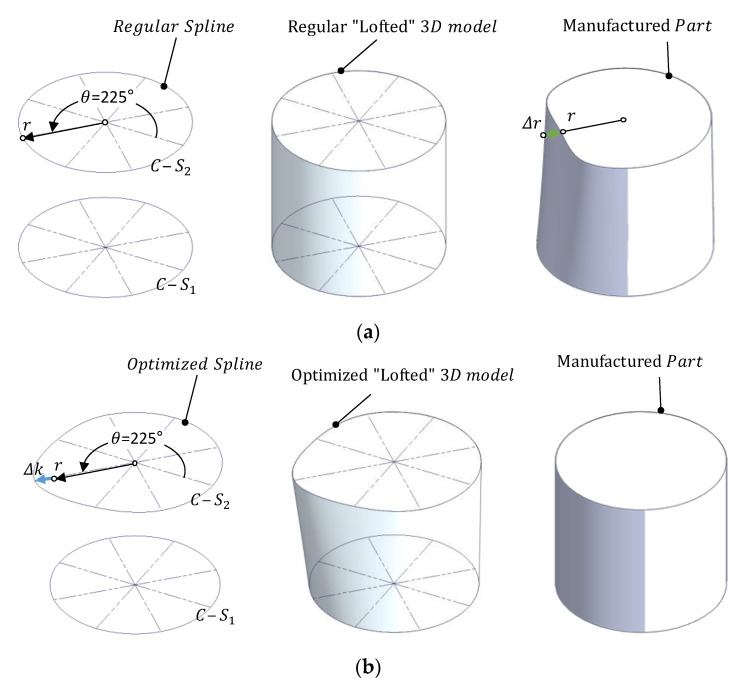
An example of 3D model generation with the loft operation and the expected results after manufacturing: (**a**) Based on regular non-distorted splines; (**b**) based on the calculated compensation.

In a CAD system, the re-parameterized cylinder will be originally constructed by using a loft operation from a combination of cross-sections and generatrixes, which have been created from bi-dimensional splines parameterized by means of the radial distance between each node and the axis (Figure 7a).

In the example of Figure 7a, a cylinder was constructed using two regular splines whose control nodes were placed exactly at the value of the nominal radius (*r*) with respect to the center of the correspondent cross-sections, that was also located at a certain *z* along the theoretical axis. A loft operation was used to generate a solid model that matches the mentioned splines. Once manufactured, the verification procedure shows that all the points are correctly located with the exception of the point with a 225° orientation in *C-S_2_*. This point presents a *Δr* deviation with respect to the expected position.

In Figure 7b, the position of correspondent node is modified using the *Δk* compensation and, consequently, an optimized spline is obtained. The loft operation generates an optimized 3D model, distorted to compensate the measured deviation. When this new design is manufactured, the expected results should be a part with a reduced dimensional and geometrical error.

The procedure finishes with the manufacturing of a series of verification trays and the evaluation of the achieved quality improvement. This analysis shall be made through the variation of each quality indicator *Δ*(*QI*) between the corresponding value calculated upon parts manufactured with the original design and the value calculated with the optimized design, e.g., the variation of size deviation (*Δ*(*ΔS*)) shall be calculated as the difference between the absolute mean size deviation for the original design ($\left|\bar{\Delta S_{or}}\right|$) and the absolute mean size deviation calculated for the optimized design ($\left|\bar{\Delta S_{op}}\right|$) (Equation (4)).

(4) $$\Delta \left(\Delta S\right)=\left|\bar{\Delta S_{or}}\right|-\left|\bar{\Delta S_{op}}\right|$$

Improvements in quality will cause positive variations of *Δ*(*ΔS*), whereas negative variations would imply that the quality has worsened. The variation of other QI would be calculated accordingly, and the results shall be interpreted in the same way.

## 3. Application Example

An application example of the proposed DfAM strategy is provided in this section. Since the methodology has already been explained in the previous section, materials and equipment are described in first place. Then, each successive step is presented and discussed independently. Results are presented along with a short discussion on the benefits and drawbacks of the strategy. 

### 3.1. Materials and Equipment

A cylindrical hollow part was selected for this application example. This part was formed by two coaxial cylindrical surfaces defined by their nominal sizes (*S_N_*) or diameters, one external (*S_N_* = 35 mm) and the other internal (*S_N_* = 30 mm). All test specimens in this work were manufactured in a Stratasys^®^ Objet 30. An acrylic photo-polymer from the Vero™ family (Stratasys VeroBlackPlus) was selected as model material whereas Stratasys FullCure 705 was chosen for supports. The Objet 30 machine used for the experiments exclusively processes some materials of the Vero™ family, which basically differ in their optical properties. The physical properties of the VeroBlackPlus are provided in Table 1.

Parts were manufactured using a glossy finish because it provides lower surface roughness than the alternative matte finish [50]. The manufacturing configuration was set to default values, following the recommendations provided by Stratasys^®^ for our Objet 30 machine, which included a 28 µm layer height and 73 °C for both model and support materials during the jetting operation. 

Manufactured specimens were verified in a DEA Global Image 09-15-08 Coordinate Measurement Machine (CMM). This machine was calibrated according to ISO 10360-2:2009 [54], and the maximum permissible error of length measurement (*E*_0,*MPE*_) was (Equation (5)):

(5) $$E_{0,MPE}(µm)=\;2.2\;+\;0.003\cdot L\;\;\;\;\;\;\;\left(L\;in\;\mathrm{mm}\right)$$

while the maximum permissible limit of the repeatability range (*R*_0,*MPL*_) was 2.2 µm. 

Metrological operations were performed using PC-DMIS metrology software. Temperature in the laboratory during verification procedures was maintained within a range of 20 ± 2 °C. Fourier analysis was performed using MATLAB^®^, while DOE and ANOVA were carried out with Minitab^®^. Finally, parts were designed using Solid Edge^®^.

### 3.2. Verification and Re-Parameterization Strategy

**Figure 8:**
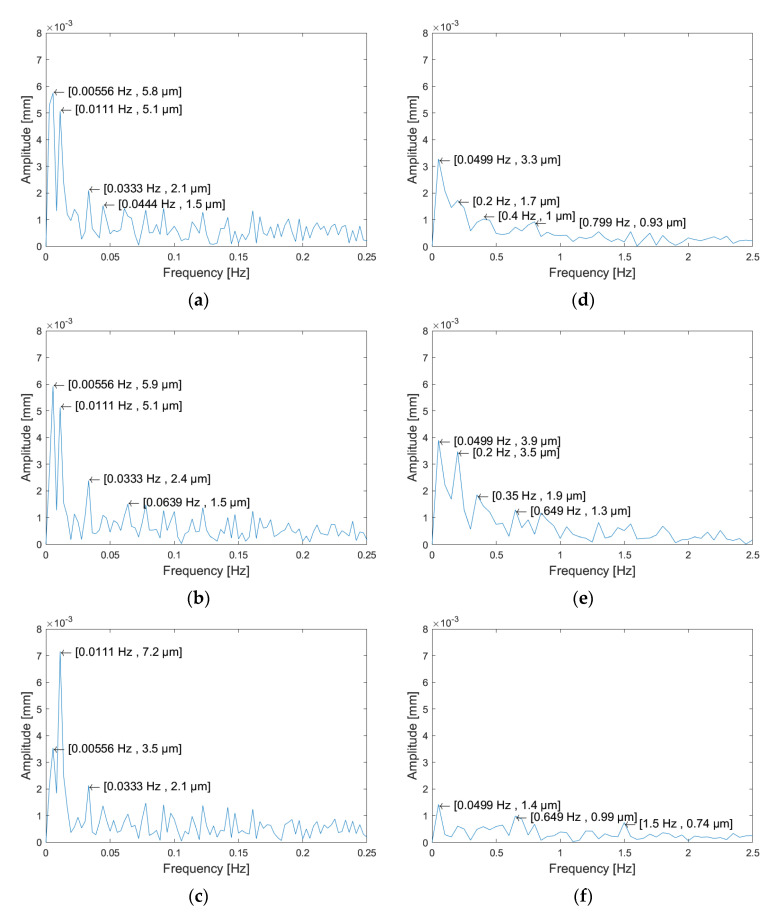
Results of the fast Fourier transform (FFT) for local deviations. Cross-sections: (**a**) z=10 mm; (**b**) z=20 mm; (**c**) z=30 mm. Generatrixes: (**d**) φ=0°; (e) φ=120°; (f) φ=240°.

Deviation analysis should provide the information required to determine a proper verification and re-parameterization strategy. A test artefact consisting of a cylinder of diameter 35mm with a hollow square through-hole used to line up the part was manufactured and measured with a continuous contact Renishaw^®^ SP25M probe. A birdcage strategy was used to digitize three cross-sections and three generatrixes. Sampling rates and stylus tip radius were independently selected for cross-sections and generatrixes, following, respectively, the recommendations provided by ISO 12181-2:2011[55] and ISO 12780-2:2011 [56]. Cross-sections were digitized at three different heights covering a 20 mm length using a dense scanning strategy, taking 3600 points. Consequently, the angular distance between adjacent digitized points was 0.1°, which allowed the characterization of undulations up to 1800 UPR. An approximate length of 20 mm was digitized along three generatrixes (0°; 120°; 240°) using an approximate distance of 0.020 mm between adjacent points. Using the digitized points, a LSRC was constructed. Finally, six independent sets of points, that contained the measured radial distances between each point and the axis of the correspondent LSRC were obtained. The average value of radial distances within each set of points was calculated and subtracted to limit the analysis in the frequency-domain to local shape deviations and avoid including the effect of size deviations related to the shrinkage phenomenon. Resulting values were then processed with a filter of outliers before applying the FFT. Independent frequency-domain decompositions are presented in Figure 8.

Undulations in cross-sections were very similar between different cross-sections, and they accounted for a notable portion of local form deviations. The main frequency component (0.00556 Hz) approximately corresponds to a period of 180°, and several harmonic components can also be observed at periods 0.0111 Hz (period of 90°) and 0.0333 Hz (period of 30°). Whereas main components showed relatively high amplitudes (up to 7.2 µm), no frequency component over 0.0333 Hz reached a 2 µm amplitude. 

The frequency components calculated for the generatrixes, on the other hand, presented some clear differences, which suggested that they were not as homogeneous as in the case of cross-sections. The main component (0.05 Hz) corresponded to a period of 20mm. As this was the approximate value of the sampling length, this indicates that the main form deviation registered along the generatrix could be modelled using few points. Nevertheless, the relative relevance of this component was different between generatrixes: Clearly dominant in the first and third ones with different amplitudes (3.3 µm in the first case and 1.4 µm in the third), but less dominant in the second generatrix where it reached a 3.9 µm amplitude against a 3.5 µm of one of his harmonics (0.2 Hz). 

Considering simultaneously the results obtained for cross-sections and generatrixes, it was clear that cross-sections had a much more relevant effect upon form deviations. Accordingly, an appropriate verification of form deviation could be based on the roundness profile extraction strategy proposed in ISO 12180-2:11 [53] and, consequently, a cross-section-based re-parameterization, with several cross-sections equally spaced along the axis could be used. To completely define both verification and parametrization, the number of control points in each cross-section, as well as the number of cross-sections, should be determined. Given that MPE_E_ was expected to be lower than 2.3 µm when measuring the radial distances, only those frequency components that showed higher amplitudes for all cross-sections were further considered.

**Figure 9:**
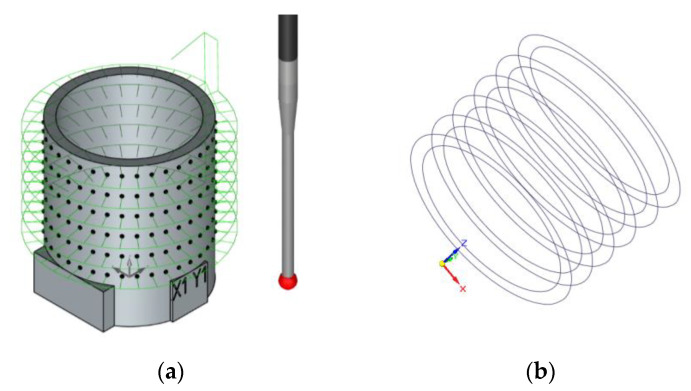
(**a**) Digitizing strategy; (**b**) cross-sections in the re-parameterized design.

Accordingly, *f_c_* for cross-sections was 0.0333 Hz, and an adequate sampling period should be lower than 15° to match the Nyquist criterion. In the present work, it was decided that a 10° sampling strategy (36 points per section) would be used to digitize cross-sections. Applying the same criterion to the generatrixes, the frequency content in that direction would be neglected. Nevertheless, low frequency components pointed out that a certain variation along the direction of the axis could be observed. Considering the digitized length in the test specimen, a minimum sampling period should be shorter than 10 mm. In the present research, a 5 mm sampling strategy has been adopted, and verification was based on six cross-sections covering 30 mm (Figure 9a).

Transposing this verification strategy to the re-parameterization of cylinders, cylindrical features in the present example would be constructed using the loft command and six parallel sketches 5 mm apart along the *Z* axis (Figure 9b). Each sketch was a closed spline with 36 nodes 10° apart, parameterized using the distance of each individual point to the origin (the intersection between the axis of the cylinder and the plane of the sketch).

### 3.3. Deviation Modelling and Design Optimization

The initial quality assessment and the analysis of model extension were conceived as concurrent tasks. The objective of this analysis was to determine, with the minimum experimental cost, if a single distortion model could be applied to all cylindrical features in a manufacturing batch or if, on the contrary, there were factors that presented a significant influence on geometrical distortion. If this was the case, factor-dependent distortion models should be applied to each combination of significant factors. Since the contact between the model material and the support material causes matte finish, glossy cylindrical surfaces can only be manufactured unsupported (with the cylinder axis parallel to the *Z* axis) [5]. Accordingly, within the limits of the proposed example, part location in the manufacturing tray was the only processing decision left to be included in the analysis of model extension. Additionally, as it was mentioned before, the default configuration of process parameters determined by Stratasys for the VeroBlackPlus was used for part manufacturing, and consequently not included in the DOE.

**Figure 10:**
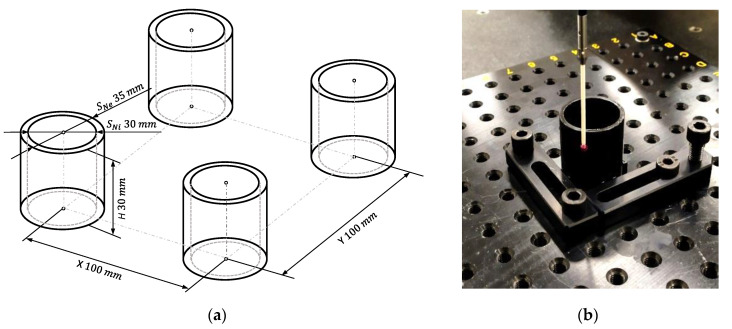
(**a**) Disposition of test specimen in the tray. (**b**) Measurement of a test specimen in the Coordinate Measurement Machine (CMM).

Consequently, the basic DOE for optimization of hollow glossy PolyJet cylinders included three factors: Two positioning factors (location along *X* axis and location along *Y* axis) and one design factor (the type of cylindrical surface –external/internal–). A 2-level full factorial design (2^3^) was selected for this example and two blocked replicates were considered to eliminate nuisance factors and account for possible variability between different manufacturing trays. No center points were included in this DOE, since it was only conceived for factor screening. Distance between parts in the tray was set to 100 mm along both *X* and *Y* directions (Figure 10a). Accordingly, eight specimens were manufactured in two different trays containing a total of 16 cylindrical features (8 external and 8 internal). Quality assessment was based on three parameters: Size deviation (*∆S*), cylindricity (j), and coaxiality (a).

Coaxiality was considered since the proposed example included two ideally coaxial cylinders in a single part. It was determined by calculating both external and internal LSRC, establishing the axis of the external LSRC as the required datum, and evaluating the coaxiality of the internal LSRC axis with respect to the datum.

Once manufactured, test specimens were measured following the defined strategy. For each cylindrical surface, a filter of outliers was applied, and then correspondent LSRCs and QI (*∆S*, j and a) were calculated. Results are provided in Table 2.

Results showed that the measured diameter of external cylinders was always bigger than the nominal ones, whereas internal cylinders showed exactly the opposite behavior, and measured diameters were lower than the nominal ones. The average result of the size deviation in this initial quality assessment was 0.040 mm in the case of the external cylinders and −0.046 mm in the case of the internal cylinders. Several previous works pointed out that PolyJet parts were subjected to an oversizing phenomenon [5,48] that was also dependent on part dimensions [52]. The fact that inner surfaces were not equally oversized was in accordance with the previous results [12,51], although the opposite behavior was reported by Maurya [48]. The main consequence of this phenomenon was that the scaling compensation at the computer-aided manufacturing (CAM) stage should not be considered a suitable strategy for dimensional improvement of PolyJet parts, at least when the design simultaneously comprises internal and external features. 

ANOVA results for *∆S* (Table 3) bear out the significance of the type of feature (*p*-value < 0.05), and this significance was also observed for part location along both X and Y axes. The Fisher’s statistical test (F-value) showed that the linear effects of the model accounted for a high significance regarding the difference of means. This significance was also found for two-way interactions, although in this case not all the terms independently showed significance. In fact, X-Y and Y-Type interactions showed a significant influence upon variance according to their *p*-value, but this was not the case of X-Type interaction.

Third order interaction was found to have no significant effect on the variability of *∆S*. Additionally, the F-value indicated that there were no statistically significant differences between blocks, meaning that the null hypothesis could not be rejected because means between the manufactured trays were not significantly different. Accordingly, since the ANOVA showed no significant dependence related to replicates (*p*-value = 1.000), this would imply that the actual size of an external feature located in X1Y1 manufactured in the first tray would not be significantly different than the actual size of the same feature manufactured in the second tray. This result pointed out that actual sizes of parts located in the same position are repeatable between trays, which is a relevant question, since otherwise it would had compromised the possibilities of applying a corrective model. On the other hand, observed variations in part size showed a significant dependence with variations in X, Y, and Type. This would mean that the actual size of the mentioned feature would show significant differences with respect to the sizes of every other feature manufactured in the same tray. 

**Figure 11:**
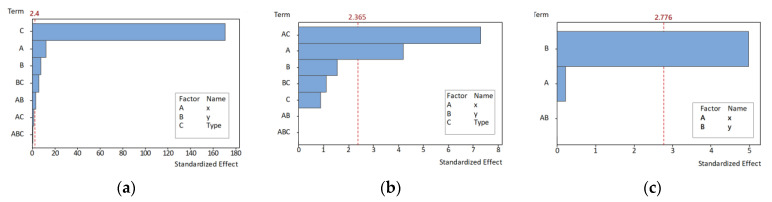
Pareto charts of the standardized effects: (**a**) Size deviation; (**b**) cylindricity; (**c**) coaxiality.

In accordance with these results, it was concluded that all the factors considered in this experiment have a significant influence upon the variability of the measured size. Consequently, individual deviation models should be defined for each combination of X, Y, and Type factors. This conclusion implied that analysis of cylindricity variance was no longer needed for the purpose of determining model extension. Nevertheless, the correspondent ANOVA showed that the observed variability in cylindricity results was mainly related to the location of each par along X direction and (even more significantly) to the interaction between such location and the type of feature. The remaining factors (location along Y direction and its interactions of second a third order) were found to have no significant effect upon cylindricity variability. Finally, the location of the part along the Y axis showed a significant influence upon coaxiality, whereas the location along the X axis was not relevant. The average cylindricity value for the optimized design was 0.036 mm in the case of external cylinders and 0.038 mm in the case of internal ones. Finally, the average result of the coaxiality was 0.022 mm. Pareto charts for size deviation, cylindricity, and coaxiality are also provided in Figure 11.

The deviation mapping task was performed using data obtained during quality assessment. The axis of the external LSRC was used as the reference axis for each part, and the radial distance of each digitized point with respect to that axis was calculated, either for external or internal features. Deviation mapping used the local radial deviation *Δr* calculated as the difference between the measured radial distance and the nominal radius of the cylinder. Since two identical trays were manufactured and measured, compensation coefficient (*ΔK*) was calculated as the average of two independent samples for each control node. Consequently, 432 deviation values, distributed in two samples, six cross-sections per part, and 36 nodes per section were calculated for each of the four locations in the tray. Following the proposed approach, radial distance *r* of each node to the reference axis was linearly modified to compensate local deviation, and a new optimized radial distance (*r_o_ = r + ΔK*) was therefore calculated.

Once this procedure was completed for all of the nodes in each model, optimized designs were generated using the loft command. The compensation values where uploaded in the table of variables of the re-parameterized design and the optimized 3D models were generated following the basic scheme presented in Figure 7. Then, each optimized model was placed in its correspondent relative location within the tray and a new set of parts were manufactured and measured. Notice that, after compensation, four different optimized CAD designs (one for each location) were used instead of a single design (common for all locations). Verification results obtained from the optimized evaluation set are provided in Table 4.

The average result of the size deviation for the optimized parts was −0.005 mm in the case of the external cylinders. Average size deviation was −0.006 mm for optimized internal cylinders. A significant reduction in size deviation has been achieved in both cases, since *Δ*(*ΔS*) = 0.035 mm for external cylinders and *Δ*(*ΔS*) = 0.040 mm for internal ones. The average cylindricity value for the optimized design was 0.031 mm in the case of external cylinders and 0.038 mm in the case of internal ones. This means that *Δ(*j*)* was 0.005 mm for external cylinders and 0.002 mm for internal ones. Accordingly, the reduction achieved for this parameter was not so significant as it was for size deviation. Finally, the average result of the coaxiality was 0.009 mm, which implies that *Δ(*a*)* was 0.013 mm. These results showed that all considered QI were improved after the application of the proposed DfMA.

In order to compare the results provided by the proposed strategy with those achieve through a conventional “shrinkage” compensation, an additional test was carried out. Two pairs of right rectangular prisms disposed along X and Y directions, respectively, were manufactured and measured. Those specimens have a nominal length of 35 mm. Once manufactured, it was found that the specimens disposed along X had an average measured length of 35.038 mm, whereas those specimens disposed along Y had an average measured length of 35.024 mm. This means that parts present an oversize phenomenon in accordance with previous findings [5,48,52]. Consequently, parts were scaled to 99.891% in the X direction and to 99.931% in the Y direction. Two new trays of cylinders with the scaled design were manufactured and measured. 

The average result of the size deviation for the optimized parts was −0.008 mm in the case of the external cylinders. Average size deviation was −0.087 mm for optimized internal cylinders. While a significant reduction in size deviation has been achieved for external cylinders (*Δ*(*ΔS*) = 0.032 mm), size deviation was increased for internal ones (*Δ*(*ΔS*) = −0.041 mm), which implies a significant worsening. The average cylindricity value for the scaled design was 0.041 mm in the case of external cylinders and 0.037 mm in the case of internal ones. This means that *Δ(*j*)* was −0.005 mm for external cylinders and 0.001 mm for internal ones. Finally, the average result of the coaxiality was 0.023 mm, which implies that *Δ(*a*)* was −0.001 mm. As it was expected, the scaling compensation strategy did not achieve any significant improvement regarding geometrical quality indicators, whereas it was also inadequate for simultaneously improving external and internal features. 

**Figure 12:**
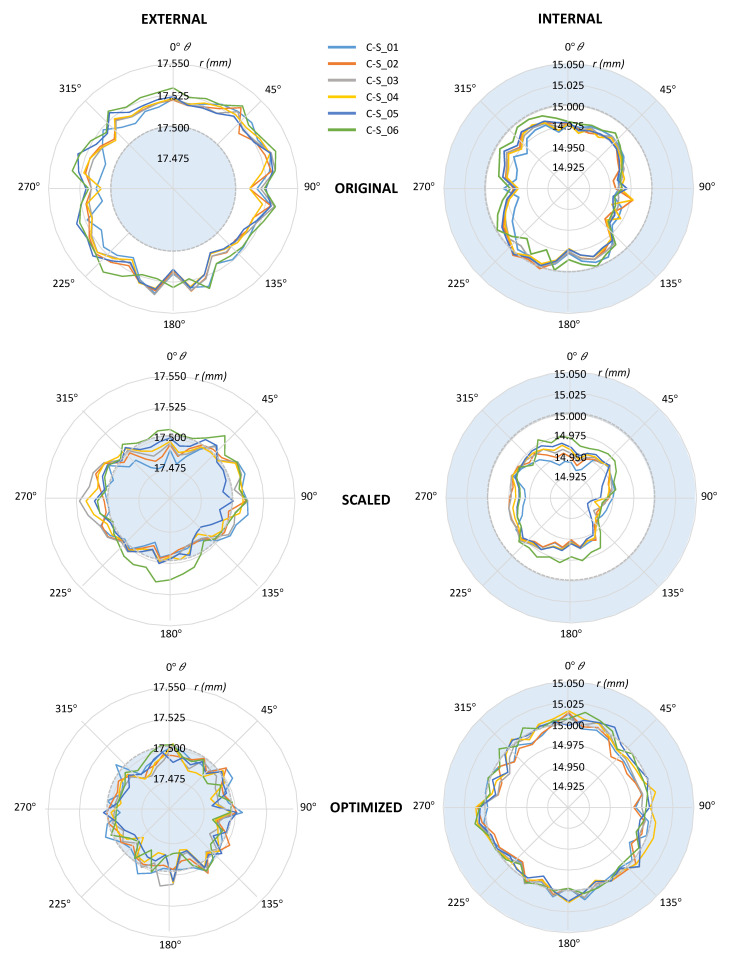
Polar graphs of the average radial deviations of digitized points with respect to the external least squares reference cylinder (LSRC) axis, calculated for parts in location X1Y1 and corresponding to the original, scaled, and optimized designs. In each graph, six cross-sections are represented, C-S_01 being the lowest and C-S_06 the highest.

To illustrate the advantages of the proposed DfAM strategy, polar graphs in Figure 12 reflect the averaged radial distances (*r*) of parts located in position X1Y1 for each test run: Original, scaled, and optimized. The radial distances were calculated with respect to the external LSRC axis, following previous conventions. Radial distances measured for the external cylinder of original design were higher than the nominal with independence of radial and axial position but, as expected, deviation was not uniform.

Deviations along the 45–225° direction were slightly higher than those along the 135–315° direction. Moreover, a tendency to higher deviations corresponding to higher Z-located sections could be observed when comparing the lowest section (C-S_01: Clear blue line) with the highest Z one (C-S_06: Green line). Nevertheless, even this behavior was not regular, and there were certain points where this general description was not accurate. In the case of the internal cylinder, deviations were clearly lower than the nominal size but, in this case, the effect was far less pronounced along the 45–225° direction, and clearly higher along the 0–180° direction. Polar graphs evidence that a common deformation model cannot be applied to external and internal surfaces simultaneously, in accordance with the ANOVA. These graphs also illustrate the lack of coaxiality between both surfaces.

Regarding the scaled design, the radial distances were closer to the nominal ones for the external cylinder, whereas negative deviations were significantly increased for the internal one. The differences between sections as well as the dominant orientations were still evident. The same occurred with the lack of coaxiality. Consequently, the scaled model was found to be inadequate, since it did not consider the local differences in deviation within cross-sections, the slight deviation along generatrixes, the significance of part position in the tray, or the relationship between axial references for both the external and the internal features.

The results obtained applying the DfAM strategy were clearly better, since the model was capable of minimizing size deviations in both cylinders, reducing the radial distances for external points and increasing them for internal points. Moreover, the cross-sections were more uniform and the tendency of upper sections to increase radial distances with respect to lower sections was also minimized. Finally, comparing deviations for different angular positions, a higher uniformity could also be observed, as hypothetical section centers were closer to the reference axis. This result is consequent with the observed reduction in coaxiality values.

### 3.4. Discussion

The main benefits of the proposed approach were:

- It allowed for a clear improvement in part quality.
- It was found to be more robust than the scaling compensation strategy.
- It also has lower complexity: It can be implemented in a conventional CAD software and does not require dense digitizing, model adjustment, or interpolation procedures.

Results showed that QI were closer to their theoretical objective values in parts manufactured with the optimized design than in parts manufactured with the original design. Instead of using a single parameter (the diameter) for the CAD optimization, the proposed strategy used the radial distances between each point and the axis of the LSRC, and this re-parameterization of the design allows to apply local compensations to the radial deviation. This leads to a significant closeness between the profile of the cross-sections and the theoretical objective (Figure 12). The aggregate effect of radial compensations results in a significant reduction of the absolute average size deviation between the original and the optimized designs. Another relevant aspect of the proposed re-parameterization was that the radial deviations were calculated for both cylinders establishing the axis of the external LSRC as the reference. Using this re-parameterization, the reduction of coaxiality (0.009 mm for the optimized design against 0.022 mm for original design) was also noticeable. This achievement was only possible because a common reference was used for measuring radial deviations. The proposed strategy provided a successful approach that is also easier to implement. Finally, reduction of cylindricity was not so equally significant. Since cylindricity evaluates the radial difference between two coaxial cylinders enclosing the actual cylindrical surface and having the least radial separation, it was highly conditioned by extreme values of the local radial differences. 

The DfAM strategy provided an optimized design that was capable of equally dealing with size deviation for both internal and external surfaces, since the ANOVA identified the Type of feature as a significant influence factor. Conversely, the scaling compensation strategy has proved to be incapable of correctly addressing this problem, since scale factors calculated for “shaft” features were only adequate for external (“shaft”) cylinders (*Δ*(*ΔS*) = 0.032 mm), whereas they worsened the size deviation of internal (“hole”) features (*Δ*(*ΔS*) = −0.041 mm). This circumstance was not described or mentioned in previous optimization efforts [52]. Additionally, since the scaling compensation strategy did not take into account relationships between different features in the same part, it was not able to reduce the coaxiality (*Δ(*a*)* = −0.001 mm). Accordingly, the proposed DfAM strategy was more adequate than the conventional scaling compensation. 

As a difference with previous research studies [16,29,37,39,42], the achieved improvements did not require dense digitizing, neither required of deviation adjustment models [29] or interpolation procedures [28,42] to accurately map form errors. Instead, a bi-univocal correspondence between verification points and design parameterization has proven to be a valid option. Moreover, optimization procedures should not assume form deviation to be independent of design and processing parameters by default. Although this kind of simplification could still allow for quality improvement under certain circumstances, it has been proved that a significance analysis of design and processing factors upon the variability of QI would help to define compensation models more accurately. On the other hand, if many significant factors were to be considered, the complexity of compensation models as well as the experimental effort will increase accordingly. The example presented here does not contemplate an a priori quality specification, since it was intended to illustrate the proposed DfAM approach for a particular combination of design, material, processing technology, and surface finish. Nevertheless, designers and manufactures should be encouraged to strictly define quality requirements and process capabilities, since the continuous improvement of AM processes should derive in manufactured parts directly fulfilling specifications without requiring optimization procedures.

Based on these results, a modified design workflow can be proposed. Designers should identify FoLS and their correspondent tolerances in the original design. Then, based on previous knowledge or extracting information from pre-series, a re-parameterization of those FoLS whose tolerances were not fulfilled should be carried out. Finally, a compensation step based on deviation mapping should be performed, and optimized quality evaluated. Although quality cannot not be improved to a zero-error level, carrying out an DfAM optimization could help to improve quality in medium-to-large production batches.

Nevertheless, the proposed DfAM strategy also has some drawbacks. Firstly, it is not adequate for manufacturing single units or small batch sizes, unless the added value justifies the experimental effort. Despite this, only two trays were required to analyze the problem and create a compensation model that significantly reduced size deviation. This experimental effort seems to be adequate for medium-to-large production batches, and comparable to the adjustment effort required in other conventional manufacturing processes. In the same way, increasing the number of replicates may increase the predictive accuracy of the deviation mapping, but the DOE is basically used in this approach for screening purposes, and this objective does not demand multiple replicates. This philosophy is in accordance with the decision of keeping the experimental cost to a minimum. Finally, no effort has been conducted to determine if a global form deviation model could be applied, considering local deviations as a function of part location, type of surface, and polar coordinates in a single model, instead of using independent models for each combination of significant factors. This possibility could lead to a general compensation model, in the direction pointed out by previous works [32,33]. Although this idea falls out of the scope of present research, it is believed to be worthy of further research. It must be noted that the proposed strategy has been tailored to improve the quality of cylinders, since they are the most frequent FoLS used for fitting. In the case of parts with parallel faces, the research team consider that a local cartesian coordinate system would be preferable to the polar one used for cylinders. This option would be also explored in further research. Finally, the proposed methodology has not already been tested for different AM processes or materials. Since geometrical optimization has a physical limit related to repeatability and stochastic errors, people in charge of the optimization effort should carefully analyze existent know-how before considering the possibility of applying the proposed DfAM strategy for situations out of the scope of this research. Including additional factors (e.g., type of material or layer height) to extend the range of application of this strategy would imply their inclusion in the DOE, so they can be analyzed through the ANOVA to determine if they have a significant influence upon size variability. If a particular application demands the modification of the recommended process configuration, each process factor that is going to be subjected to modifications should be also included in the DOE. 

## 4. Conclusions

This work presents a design for additive manufacturing strategy that has been tailored to improve dimensional and geometrical quality of cylindrical features manufactured in a glossy finish with the PolyJet MJT technology. The fast Fourier transform is used to analyze the components of form deviations in the frequency domain, and determine an adequate verification strategy. A design re-parameterization based on a bi-univocal correspondence between verification parameters and design parameters is then performed. In a second step, the relevance of process and design factors upon the variability of quality indicators is analyzed using design of experiments and ANOVA. An adequate model extension is therefore determined to include those factors that show significant influence upon the responses. Local deviations from the exact shape are then used to optimize the 3D design. Deviation mapping allows us to apply local compensations to the re-parameterized design according to model extension. Geometrical restrictions, like the relative position or the coaxiality, can be also included in the model. The proposed DfAM strategy has several advantages. Firstly, it can be implemented in a conventional 3D design software package, instead of demanding specific programming. Secondly, it allows for particularizing compensation models for combinations of relevant influence factors. Thirdly, it avoids the necessity of adjusting deformation models to large sets of data or applying interpolation procedures to relate part deformation to CAD compensation. An application example, aiming at dimensional improvement of glossy cylindrical surfaces manufactured in a PolyJet Object 30 machine, endorses the usefulness of the proposed approach, since a significant reduction of size deviation has been achieved. Moreover, geometrical quality indicators, like cylindricity or coaxiality, were also improved. This strategy has showed better results than conventional scaling compensation strategies, since these do not properly account for deviations when the variability of quality indicators is affected by influence factors like the type of feature.

## Figures and Tables

**Table 1:** Physical properties of VeroBlackPlus.

Property	Test Method	Value
Color		Black
Tensile Strength	ASTM D638	58 MPa
Elongation at Break	ASTM D638	10–25%
Modulus of Elasticity	ASTM D638	2500 MPa
Flexural Strength	ASTM D790	93 MPa
Flexural Modulus	ASTM D790	2700 MPa
Shore D Hardness		85 D
Tensile Strength	ASTM D638	58 MPa
Heat Deflection Temperature	ASTM D648 @ 264 psi	48 °C

**Table 2:** Full factorial design of experiment (DOE) structure and quality indicator (QI) results obtained during quality assessment.

Run Order	Block	X	Y	Type	*∆S* [mm]	j [mm]	a [mm]
1	1	1	1	External	0.046	0.030	
2	1	1	2	External	0.039	0.044	
3	1	2	1	External	0.037	0.032	
4	1	2	2	External	0.034	0.042	
5	1	1	1	Internal	−0.041	0.041	0.031
6	1	1	2	Internal	−0.049	0.035	0.015
7	1	2	1	Internal	−0.043	0.040	0.030
8	1	2	2	Internal	−0.048	0.033	0.016
9	2	1	1	External	0.047	0.029	
10	2	1	2	External	0.040	0.041	
11	2	2	1	External	0.039	0.026	
12	2	2	2	External	0.035	0.041	
13	2	1	1	Internal	−0.042	0.039	0.025
14	2	1	2	Internal	−0.050	0.038	0.019
15	2	2	1	Internal	−0.044	0.043	0.025
16	2	2	2	Internal	−0.050	0.034	0.017

**Table 3:** ANOVA of full factorial DOE for *∆S*.

Source			DF	Adj SS	Adj MS	F-Value	*p*-Value
Model			8	0.029489	0.003686	3686.09	0.000
	Blocks		1	0.000000	0.000000	0.00	1.000
	Linear		3	0.029441	0.009814	9813.75	0.000
		x	1	0.000144	0.000144	144.00	0.000
		y	1	0.000056	0.000056	56.25	0.000
		Type	1	0.029241	0.029241	29241.00	0.000
	2-Way Interactions		3	0.000047	0.000016	15.75	0.002
		x*y	1	0.000009	0.000009	9.00	0.020
		x*Type	1	0.000002	0.000002	2.25	0.177
		y*Type	1	0.000036	0.000036	36.00	0.001
	3-Way Interactions		1	0.000000	0.000000	0.25	0.632
		x*y*Type	1	0.000000	0.000000	0.25	0.632
Error							
			7	0.000007	0.000001		
Total			15	0.029496			

**Table 4:** QI results after optimization.

ID	Tray	X	Y	Type	∆S [mm]	j[mm]	a [mm]
1	1	1	1	External	−0.012	0.028	
2	1	1	2	External	−0.012	0.024	
3	1	2	1	External	−0.002	0.032	
4	1	2	2	External	−0.007	0.027	
5	1	1	1	Internal	−0.004	0.036	0.010
6	1	1	2	Internal	−0.006	0.041	0.008
7	1	2	1	Internal	−0.004	0.033	0.017
8	1	2	2	Internal	−0.003	0.037	0.008
9	2	1	1	External	−0.008	0.027	
10	2	1	2	External	−0.002	0.037	
11	2	2	1	External	0.001	0.044	
12	2	2	2	External	0.001	0.027	
13	2	1	1	Internal	−0.009	0.034	0.009
14	2	1	2	Internal	−0.009	0.039	0.008
15	2	2	1	Internal	−0.006	0.036	0.012
16	2	2	2	Internal	−0.004	0.034	0.003
